# A Method for Estimating Fluorescence Emission Spectra from the Image Data of Plant Grain and Leaves Without a Spectrometer

**DOI:** 10.3390/jimaging11020030

**Published:** 2025-01-21

**Authors:** Shoji Tominaga, Shogo Nishi, Ryo Ohtera, Hideaki Sakai

**Affiliations:** 1 Department of Computer Science, Norwegian University of Science and Technology, 2815 Gjovik, Norway; 2 Department of Business and Informatics, Nagano University, Ueda 386-1298, Japan; 3 Department of Engineering Informatics, Osaka Electro-Communication University, Neyagawa 572-8530, Japan; s-nishi@osakac.ac.jp; 4 Kobe Institute of Computing, Graduate School of Information Technology, Chuo-ku, Kobe 650-0001, Japan; ryotera@kic.ac.jp; 5 Graduate School of Informatics, Kyoto University, Kyoto 606-8501, Japan; hsakai@i.kyoto-u.ac.jp

**Keywords:** fluorescence emission spectra, spectral estimation method, multiband imaging system, plant grain and leaves, ridge regression approach, cross-validation technique

## Abstract

This study proposes a method for estimating the spectral images of fluorescence spectral distributions emitted from plant grains and leaves without using a spectrometer. We construct two types of multiband imaging systems with six channels, using ordinary off-the-shelf cameras and a UV light. A mobile phone camera is used to detect the fluorescence emission in the blue wavelength region of rice grains. For plant leaves, a small monochrome camera is used with additional optical filters to detect chlorophyll fluorescence in the red-to-far-red wavelength region. A ridge regression approach is used to obtain a reliable estimate of the spectral distribution of the fluorescence emission at each pixel point from the acquired image data. The spectral distributions can be estimated by optimally selecting the ridge parameter without statistically analyzing the fluorescence spectra. An algorithm for optimal parameter selection is developed using a cross-validation technique. In experiments using real rice grains and green leaves, the estimated fluorescence emission spectral distributions by the proposed method are compared to the direct measurements obtained with a spectroradiometer and the estimates obtained using the minimum norm estimation method. The estimated images of fluorescence emissions are presented for rice grains and green leaves. The reliability of the proposed estimation method is demonstrated.

## 1. Introduction

The use of fluorescent objects has become increasingly common in recent times because the addition of fluorescent agents to materials improves their visual appearance. Fluorescent substances are commonly found in everyday products such as paper, paint, plastics, and clothing. Fluorescence is an optical phenomenon in which a material is first excited by light radiation in a specific wavelength region; upon relaxation, the excited state emits light radiation in a longer wavelength region [1,2]. When the excitation wavelengths are in the ultraviolet region, the emission wavelengths are mostly in the visible region. Therefore, several fluorescent surfaces appear brighter and more vivid than their original appearance, based on surface light reflection. Previous studies on fluorescence analysis have mainly targeted man-made objects, such as those mentioned above ([3,4,5]), where the bispectral characteristics of fluorescent objects consisting of fluorescent emission and reflectance spectra were often estimated.

However, a variety of natural objects exist in addition to man-made ones that emit fluorescence, such as minerals, deep-sea fish, corals, and plants. Among these, plants have recently attracted significant interest as a research topic owing to the need to increase food production and security as the population increases [6,7,8,9,10]. In this study, we estimate the spectral images of fluorescence spectral distributions emitted from plant grains and leaves. Polished rice grains emit fluorescence in the blue-wavelength range. Fluorescence is emitted from lipids (fat) contained in rice, and the intensity of fluorescence increases as lipids are oxidized. After harvest, as time passes, the oxidation of lipids in rice progresses, the fluorescence intensity increases, and the freshness of rice can be predicted by estimating the fluorescence intensity (e.g., see [11]). The fluorescence spectral distribution of rice depends on its origins [12]. Therefore, the origin of rice can be identified by estimating its fluorescence spectral distribution, which has a positive impact on food safety. As a first step toward the identification of freshness and origin, we developed an imaging system and algorithm to estimate the distribution of fluorescence emitted by rice as a spectral image with high accuracy.

Plant leaves contain chlorophyll that emits fluorescence in the red-to-near-infrared wavelength range. The spectral distribution of chlorophyll varies according to the plant species and environmental conditions [13]. This phenomenon makes fluorescence spectroscopy an effective method for investigating plant growth and conditions. In this study, we consider a spectral imaging method as a basic and useful tool to perform such studies.

Specialized equipment, such as a spectroradiometer or spectrocolorimeter, is required to directly measure the spectral distribution of fluorescent emission. However, these instruments are expensive and poorly portable, and a major issue is that they can only obtain spectral information of one point in a scene. A spectral imaging system equipped with filters for a camera, such as a liquid-crystal tunable (LCT) filter, may be useful for obtaining a spectral image of a scene. Such equipment is also expensive, poorly portable, and has low resolution. In this study, we construct a multiband imaging system by adding the minimum number of necessary filters to ordinary, small off-the-shelf cameras.

**Figure 1:**
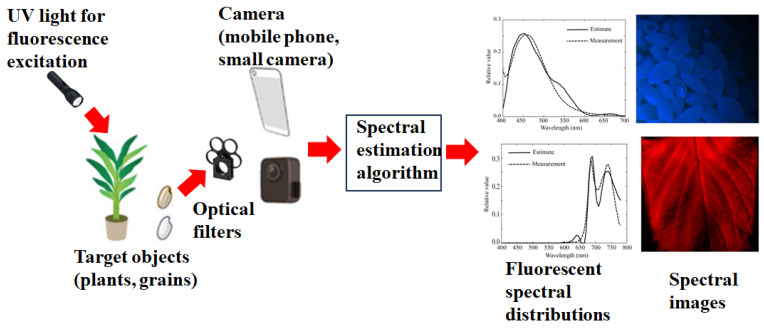
Overview of the multiband imaging system proposed for estimating the fluorescence emission spectra from plants and grains.

Figure 1 presents an overview of the proposed multiband imaging system for estimating the spectral images of fluorescence spectral distributions emitted from plants and grains. The target plant leaves or rice grains are illuminated with UV light for fluorescence excitation. The emitted fluorescent light passes through filters and is captured by a mobile phone camera or small monochrome camera. The spectral estimation algorithm estimates the spectral distribution of the fluorescence emitted at every pixel point from the captured images and outputs a spectral image. The top and bottom figures on the right of Figure 1 show examples of the emitted fluorescent spectral distributions and sRGB images converted from the spectral images of rice grains and perilla leaves, respectively. It should be noted in Figure 1 that we do not use compact equipment, such as a standard spectrofluorometer. We intend to estimate the fluorescence spectral images emitted from rice grains and plant leaves, including living leaves, outdoors. Therefore, the positions of the excitation light source, target object, and camera should not be fixed, as shown in Figure 1. The system proposed in [12] for measuring fluorescence emission distributions appears to be similar to our idea.

Numerous methods have been proposed in the fields of imaging science and technology, and computer vision for estimating spectral distributions from camera data. However, most of these methods are based solely on the reflected light from a non-fluorescent object and not on emitted light, such as fluorescence. For example, the Wiener estimation method and the recent linear minimum mean square error (LMMSE) method are well-known [14]; however, they are based solely on the reflected light from an object’s surface and estimate the spectral reflectance of the object; thus, they cannot be applied to estimate the spectral distribution of fluorescent emission. These are statistical methods that use spectral reflectance databases. The wavelength band can differ in the case of fluorescence emissions; therefore, a spectral database is not available. In our preliminary study [6], we employed a simplified Wiener-like method that did not use a fluorescence database. However, it was assumed that the spectroradiometer could be used as a ground truth.

In this study, we propose a method to obtain a reliable estimate of the fluorescence emission spectral distribution at every pixel point from only the acquired image data without relying on a spectrometer. The data measured using a spectroradiometer are used to verify the reliability of the estimation results. We adopt a ridge regression method [15,16,17], which is used for estimating the coefficients of multiple regression models in scenarios where independent variables are highly correlated. This approach does not require statistics such as the mean, variance, and autocorrelation matrix. By optimally selecting the ridge parameter, we can obtain a much more reliable estimate of the fluorescence emission spectral distribution than using the minimum norm solution.

The remainder of this paper is organized as follows. First, we introduce two types of multiband systems: one for rice grains using a mobile phone camera and the other for plant leaves using a small monochrome camera. Second, an estimation method is developed. We describe an observation model for problem formulation and propose an algorithm to optimally estimate the fluorescence emission spectral distribution from camera data. Third, in experiments, UV light is used to illuminate real rice grains and green leaves, the emitted fluorescence spectra and images are estimated, and the reliability of the proposed method is demonstrated.

## 2. Imaging Systems

### 2.1. Light Source and Spectrometer

**Figure 2:**
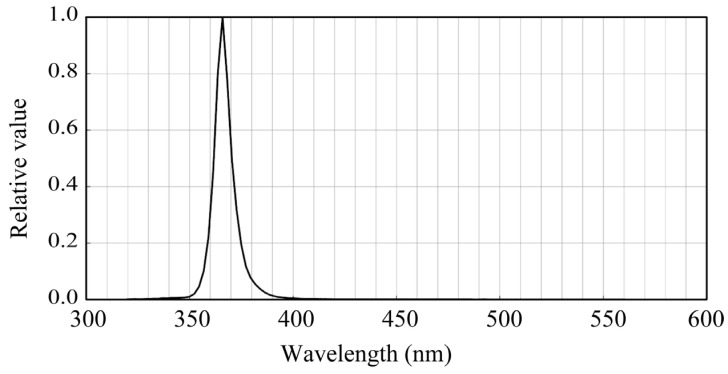
Spectral power distribution of the used UV LED light.

UV LED light (NCSU276A, NICHIA, Tokushima, Japan) was used to excite fluorescence in the plants. Figure 2 shows the spectral power distribution where the UV light peak wavelength was 365 nm. The global spectral distribution of the fluorescent light was measured using a spectroradiometer (CS-2000; Konica Minolta, Tokyo, Japan), which was also used to verify the estimated spectral distribution of the fluorescence emission.

### 2.2. Multiband System Using a Mobile Phone Camera

**Figure 3:**
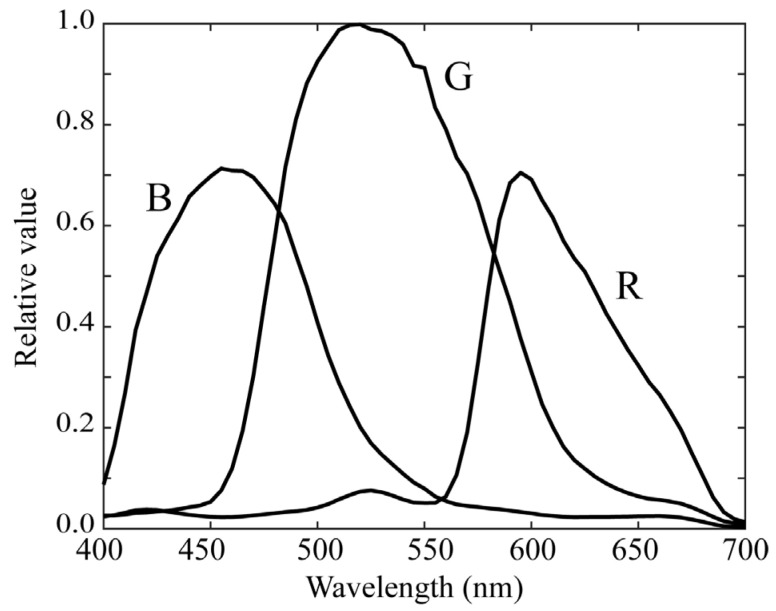
Spectral sensitivity functions of the RGB camera.

**Figure 4:**
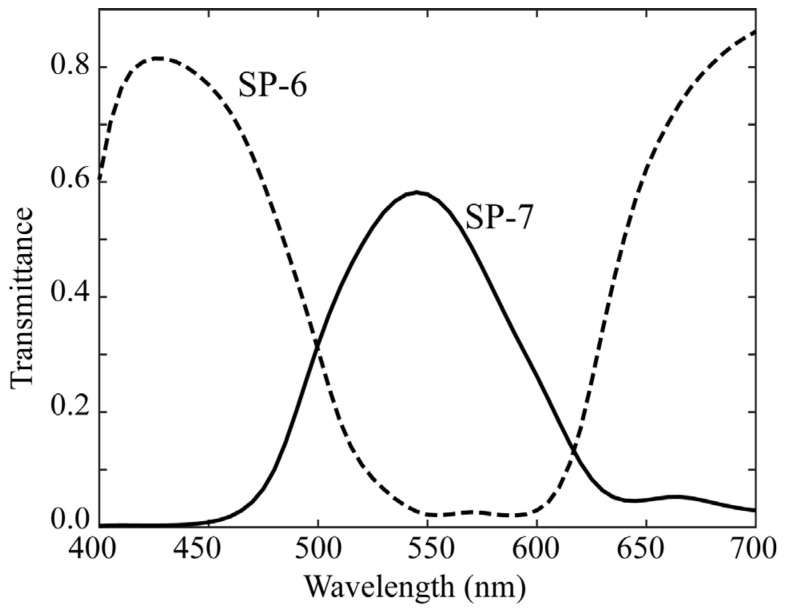
Spectral transmittance curves of the two filters used.

**Figure 5:**
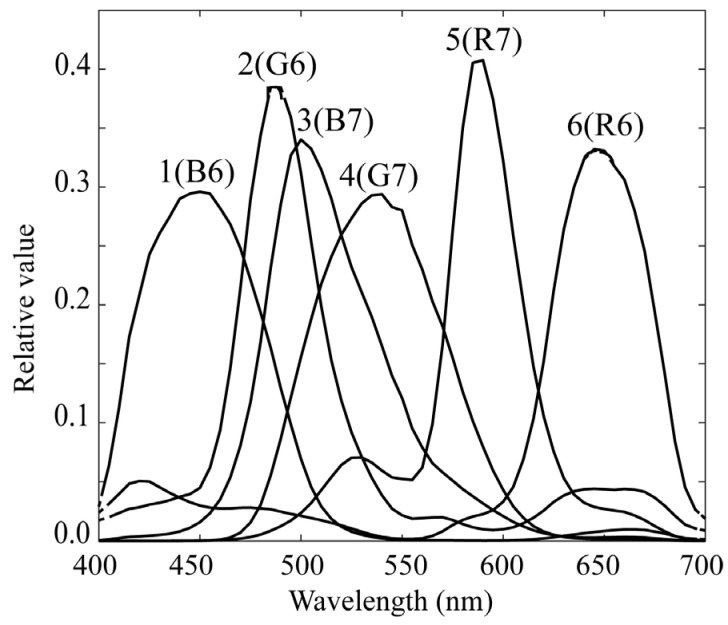
Overall spectral sensitivity functions calculated by multiplying the spectral sensitivity functions in Figure 3 and spectral transmittances in Figure 4. To clarify that the imaging system has six bands, we numbered each spectral sensitivity from the lowest wavelength.

As the fluorescence emission from lipids in rice grains has a spectral distribution mostly in the visible wavelength range, we used a mobile phone camera with RGB channels (iPhone 6s; Apple Inc., Cupertino, CA, USA) and made it multiband. A similar multiband method was proposed to calculate the surface spectral reflectance [18]. The camera depth was set to 12 bits. The curves of the spectral sensitivity functions of the RGB camera are shown in Figure 3. For multiband image acquisition, we selected two color filters from a set of commercial color filters (Fujifilm Optical Filter, Fujifilm, Tokyo, Japan). Figure 4 shows the spectral transmittance curves of the filters. Combining these transmittances with the original spectral sensitivities yields different sets of trichromatic spectral sensitivity functions. The SP-6 filter was effective in shifting the spectral sensitivity to short and long wavelengths in the visible range, whereas the SP-7 filter was effective in shifting the spectral sensitivity to middle wavelengths. Thus, two sets of modified trichromatic spectral sensitivities resulted in an imaging system with six spectral bands in the visible wavelength region. Figure 5 shows the overall spectral sensitivity functions of the proposed multiband imaging system with six channels constructed using an RGB mobile phone camera and two color filters. Notably, this multiband imaging system is suitable for fluorescence image acquisition over the entire visible range. Each filter was attached to the front of a mobile phone camera lens. The image of each channel was acquired using Adobe’s digital negative (DNG) format.

We created plastic holders for the filters, and the camera was fixed on a tripod.

### 2.3. Multiband System Using a Monochrome Camera

**Figure 6:**
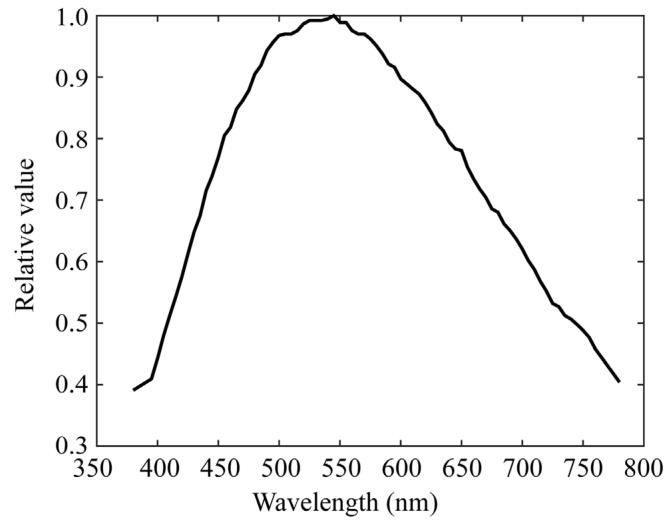
Spectral sensitivity function of the monochrome camera.

**Figure 7:**
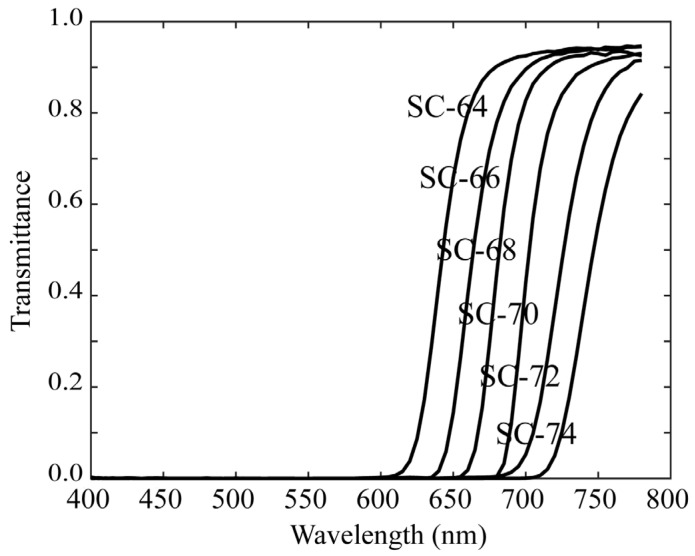
Spectral transmittance curves of the six sharp-cut filters.

**Figure 8:**
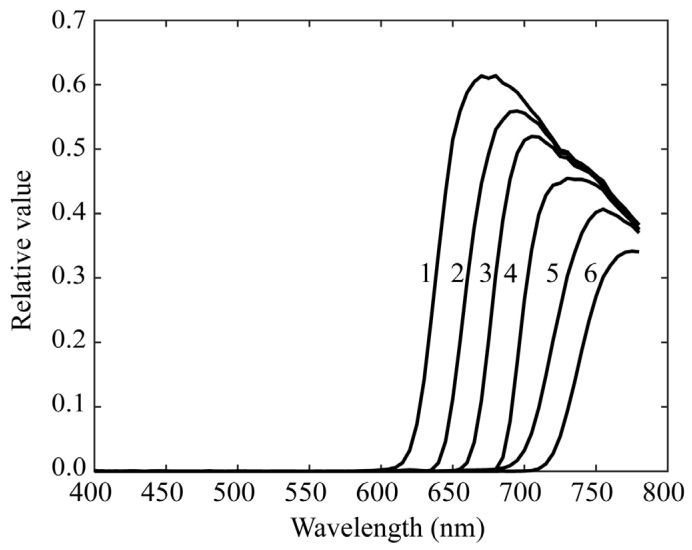
Overall spectral sensitivity functions of the multiband imaging system constructed using a monochrome camera and six sharp-cut filters.

Chlorophyll fluorescence emissions from plant leaves have a spectral distribution in the red-to-far-red wavelength regions. Because the mobile phone camera did not have sufficient sensitivity in this wavelength region, as shown in Figure 3, we used a monochrome camera and made it multiband with additional filters. The camera used was a monochrome CCD camera with a 12-bit dynamic range and a Peltier cooling (QImaging, Retiga 1300, Shoshin EM, Aichi, Japan). Additional optical filters were selected such that the combination of camera sensitivity and filter transmittance resulted in overall spectral sensitivity in the red-to-far-red region. Figure 6 shows the spectral sensitivity function of the monochrome camera, which has a sensitivity of approximately 800 nm. Figure 7 shows the spectral transmittance curves of six sharp-cut filters, SC-64, SC-66, SC-68, SC-70, SC-72, and SC-74 (Fujifilm Optical Filter, Fujifilm, Tokyo, Japan), which were selected for multiband image acquisition. Figure 8 exhibits the overall spectral sensitivity function of the multiband imaging system. For the filters, we fabricated six holders using a 3D printer and fixed the camera on a tripod.

The present imaging system is specialized for detecting fluorescence spectra in the red-to-far-red regions. The image data were acquired in 12-bit tag image file format (TIFF). The fluorescence emission from rice grains can also be detected using the monochrome camera with six appropriate filters. However, the first imaging system using a mobile phone camera with two filters is much simpler in this case.

## 3. Spectral Estimation Method

### 3.1. Observation Model

We used two types of multiband imaging systems with six channels to obtain fluorescence images emitted from rice grains and plant leaves. Let the outputs of the imaging system be expressed as six observations, $y_{i}\;$ (*i* = 1, 2, …, 6) for each pixel.

(1) $$y_{i}\;=\;\int x(\lambda )r_{i}(\lambda )d\lambda ,$$

where x(λ) denotes the spectral power distribution of the fluorescence emitted at each pixel from the target plant, and $r_{i}(\lambda )$ (*i* = 1, 2, …, 6) denotes the spectral sensitivity functions of the multiband imaging systems. The wavelength range of integration is in the visible range (400–700 nm) for the first imaging system of rice grains and in a wider range (400–780 nm), including red to far-red, for the second imaging system of plant leaves. The sensitivity functions $r_{i}(\lambda )$ presented in Figure 5 and Figure 8 correspond to the first and second systems, respectively. They are defined by multiplying the RGB spectral sensitivity functions of the mobile phone camera and the spectral transmittance of the additional color filters for the first system, and by multiplying the spectral sensitivity functions of the monochrome camera and the spectral transmittance of the additional sharp-cut filters for the second system.

For the digital representation, the fluorescence spectra and spectral sensitivities are sampled at *N* wavelength points at equal intervals and represented by *N*-dimensional column vectors as follows:

(2) $$x=\left[\begin{array}{c} x(\lambda_{1})\\ x(\lambda_{2})\\ \vdots \\ x(\lambda_{N}) \end{array}\right],\;\;\;\;\;\;\;\;\;\;\;r_{i}=\left[\begin{array}{c} r_{i}(\lambda_{1})\\ r_{i}(\lambda_{2})\\ \vdots \\ r_{i}(\lambda_{N}) \end{array}\right]\;\;\;\;\;\;\;\;\;\;\;\;(i=1,\;2,\;\ldots ,\;6).$$

The discrete representation of the observation model is expressed in matrix form as follows:

(3) y = Ax,

where **A** denotes a (6 × *N*) matrix defined by the spectral sensitivities and is expressed as follows:

(4) $$A^{t}=\;\left[r_{1},\;\;r_{2},\;\;\cdots \;\;,\;\;r_{6}\right]\Delta \lambda$$

and **y** is a 6-dimensional column vector expressed as follows:

(5) $$y=\;\left[\begin{array}{c} y_{1}\\ y_{2}\\ \vdots \\ y_{6} \end{array}\right].$$

Superscripts *t* and Δλ in Equation (4) represent the matrix transposition and wavelength sampling intervals, respectively. When the continuous spectra were sampled with Δλ = 5, the discrete spectral functions are represented by 61-dimensional column vectors with *N* = 61 in the 400–700 nm range for the first system and 77-dimensional column vectors with *N* = 77 in the 400–780 nm range for the second imaging system.

### 3.2. Estimation Algorithm

We estimate the fluorescence spectrum **x** from observation **y** at every pixel point based on the observation model in Equation (3). In a preliminary study [6], a statistical approach using the simplified Wiener-like method was presented for a model with a noise term, where the autocorrelation matrix was formally set to an identity matrix; that is, every spectrum other than itself was uncorrelated, and the noise variance was estimated as an unknown parameter. We assumed that the spectral distribution of the fluorescence emission could be measured directly and simultaneously with a spectroradiometer by capturing an image using a multiband imaging system. Therefore, the noise variance was determined to minimize the error between the average fluorescence spectra estimated over the target image area and the fluorescence spectrum measured directly by the spectroradiometer.

Herein, we consider a method to estimate the best fluorescence emission spectral distribution from only acquired image data without relying on a spectroradiometer. Let us consider the number of observations *p* (=6). For p≥N, we have the well-known least-squares solution. Here, we note that p<N. In this case, an infinite number of solutions satisfy Equation (3), and the minimum norm solution among them is expressed as follows (see [19]):

(6) $$\hat{x}=A^{t}{\left(AA^{t}\right)}^{-1}y\;\;.$$

However, this method sometimes has a large estimation error and is not reliable for solving the present problem.

In this study, we adopt the ridge regression method [15,16,17], which estimates the coefficients of multiple regression models in scenarios where the independent variables are highly correlated. Ridge regression can provide a possible solution to the imprecision of minimum norm estimators when linear regression models have highly correlated independent variables. Consequently, the following ridge regression estimator often has a smaller error than the minimum norm estimator in Equation (6).

In the present case, the ridge estimator is expressed as follows:

(7) $$\hat{x}=A^{t}{\left(AA^{t}+\alpha I_{p}\right)}^{-1}y\;\;,$$

where **y** denotes the *p*-dimensional observation vector, **A** denotes the (*p* × *N*) matrix defined in (4), and $I_{p}$ denotes the (*p* × *p*) identity matrix. The ridge parameter α *λ* ≥ 0 serves as the constant shifting the diagonals of matrix $AA^{t}$. Therefore, even if matrix $AA^{t}$ is nearly singular, the influence of the singularity is alleviated by adding constant elements to the diagonals.

To identify the most appropriate ridge parameter α, we use a cross-validation technique called *p*-fold cross-validation or leave-one-out [20]. This technique divides the data into *p* parts, one of which is used as test data, and the remaining *p* − 1 parts are used as training data to evaluate the accuracy rate. Training is performed *p* times such that all *p* pieces of data are used as test data once, and then the average accuracy is obtained. The practical algorithm is as follows:

First, we divide matrix **A** and vector **y** as follows:

(8) $$A=\left[\begin{array}{c} a_{1}^{t}\\ a_{2}^{t}\\ \vdots \\ a_{p}^{t} \end{array}\right],\;\;\;\;\;\;y=\left[\begin{array}{c} y_{1}\\ y_{2}\\ \vdots \\ y_{p} \end{array}\right]\;.\;\;\;\;$$

where $a_{i}^{t}=r_{i}^{t}\Delta \lambda$ (*i* = 1, 2, …, *p*), and then define the remainder after removing the *i*-th row from **A** and **y** as follows:

(9) $$A_{\left(i\right)}=\left[\begin{array}{c} \begin{array}{c} a_{1}^{t}\\ \vdots \\ a_{i-1}^{t} \end{array}\\ a_{i+1}^{t}\\ \vdots \\ a_{p}^{t} \end{array}\right],\;\;\;\;\;\;y_{\left(i\right)}=\left[\begin{array}{c} \begin{array}{c} y_{1}\\ \vdots \\ y_{i-1} \end{array}\\ y_{i+1}\\ \vdots \\ y_{p} \end{array}\right]\;,\;\;\;\;\;\;\;\;\;\;\;\;\;\;\;\;\;\;(i=1,\;\;2,\;\;\ldots ,\;\;\;\;p).\;\;\;\;$$

The estimate of **x** and error of the test observations using these data are described as follows:

(10) $${\hat{x}}_{i}=\;A_{\left(i\right)}^{t}{\left(A_{\left(i\right)}A_{\left(i\right)}^{t}+\alpha I_{p-1}\right)}^{-1}y_{\left(i\right)},$$

(11) $$e_{i}=y_{i}\;-\;a_{i}^{t}{\hat{x}}_{i},\;\;\;\;\;\;\;\;\;$$

where *i* = 1, 2, …, *p*. The sum of the squared errors is then obtained as follows:

(12) $$J≜\sum_{i=1}^{p}e_{i}^{2}=\sum_{i=1}^{p}{\left(y_{i}-a_{i}^{t}{\hat{x}}_{i}\right)}^{2}.$$

Therefore, the ridge parameter α is determined to minimize the error function *J.* Normally, the optimal value of α is searched in the range of α > 0.

## 4. Experimental Results

### 4.1. Fluorescence Estimation for Rice Grains

**Figure 9:**
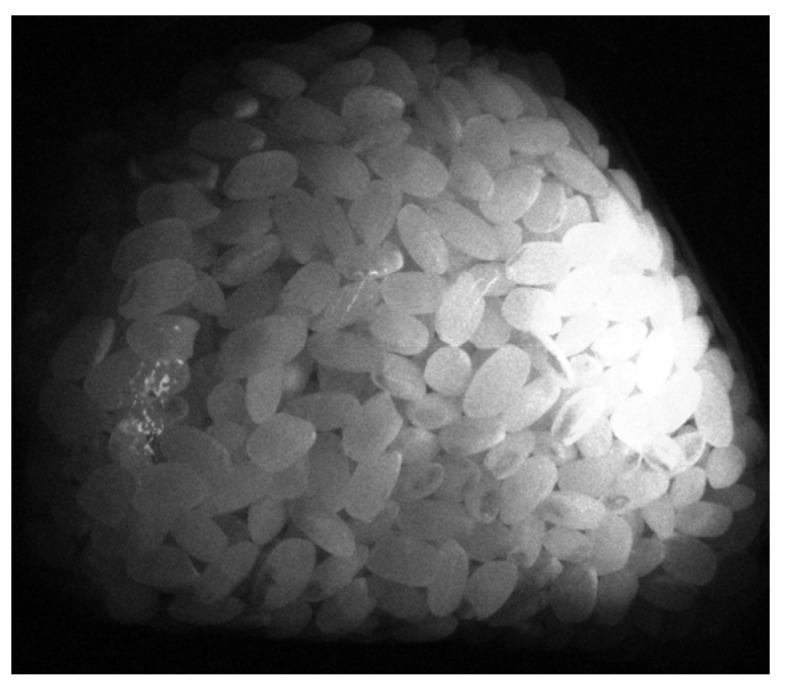
Photographic image of the rice grains used in the experiment.

**Figure 10:**
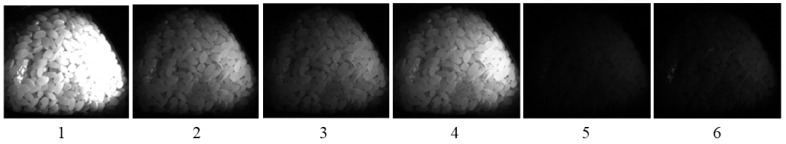
Images of each channel observed using the imaging system with six bands for the rice grains.

We examined “blended rice,” which is a mixture of rice from different regions and brands. As shown in Figure 9, the rice grains were placed in a transparent bag and illuminated with UV light from the outside. Multiband images of rice grain surfaces were captured using the first imaging system. Figure 10 shows an image of each channel observed using the imaging system with six bands. Observation **y** was averaged over an appropriate region of the object’s surface to estimate the spectral distribution of the fluorescence emission.

**Figure 11:**
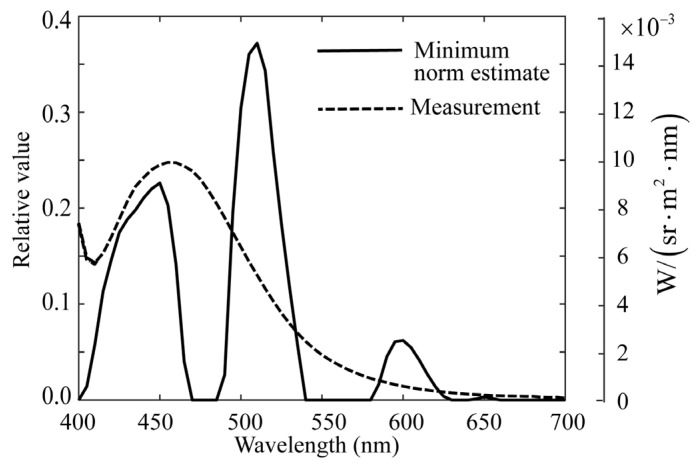
Comparison of the minimum norm estimate for the spectral distribution of fluorescent emission obtained from the image data of rice grains with the directly measured fluorescence spectrum using the spectroradiometer. To compare the estimated spectral distribution with the physical quantities measured by the spectroradiometer, we add a scale in physical quantities with the unit of $W/(\mathrm{sr}\cdot m^{2}\cdot \mathrm{nm})$ to the right in the figure.

First, the minimum norm estimate was obtained using Equation (6). Figure 11 shows this estimate, which is compared with the spectral distribution measured using a spectroradiometer. In the figure, the estimated $\hat{x}$ and measured $x_{0}$ are normalized to $\left\|\hat{x}\right\|=\left\|x_{0}\right\|=1$. The minimum norm estimate had a large sum of squared errors (0.5807) and is unreliable. The increase in the measured spectral curve at 400 nm was attributed to the effect of the illuminated UV light. To compare the estimated spectral distribution with the physical quantities measured by the spectroradiometer, we added a scale in the physical quantities with the unit $W/(\mathrm{sr}\cdot m^{2}\cdot \mathrm{nm})$ to the right of Figure 11.

**Figure 12:**
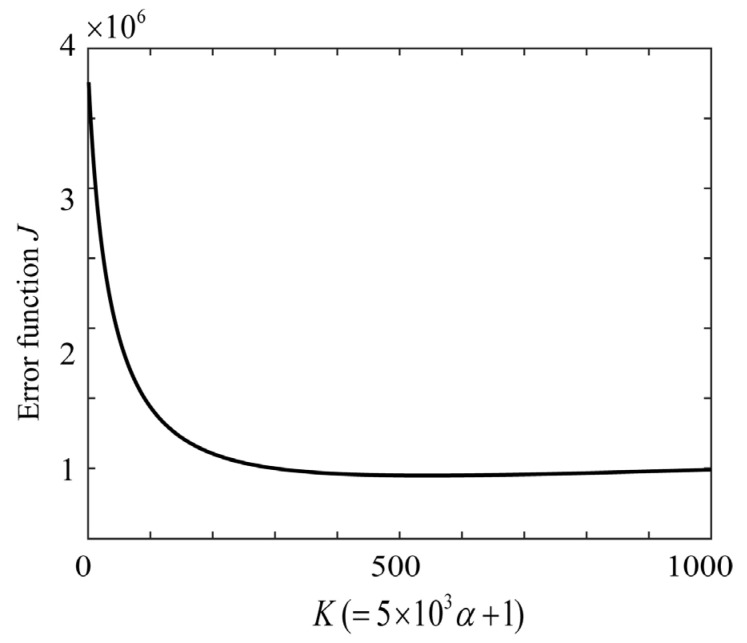
Error function *J* with *K* as a parameter to evaluate the ridge estimate for the rice grains.

**Figure 13:**
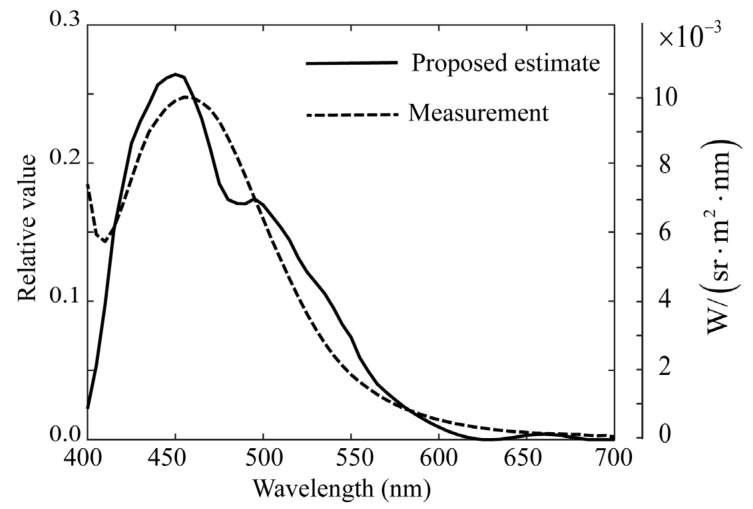
Ridge estimation result for the rice grains, where the estimated spectral curve is compared with the direct measurement using the spectroradiometer.

Subsequently, the proposed ridge estimation method was applied to the present problem. The function for evaluating the estimation is the squared sum of the prediction errors, that is, *J* defined in Equation (12). Figure 12 shows the calculated function *J*, where the parameter *K* is related to the ridge parameter α as $\alpha \;=\;2.0\times 10^{-4}(K-1)$ (*K* = 1, 2, …, 1000). We varied *K* and searched for an optimal α that minimized *J*, where *K* = 543 and α = 0.1084. Figure 13 shows the ridge estimation results, where the estimated spectral curve of $\hat{x}$ is compared with the direct measurement of $x_{0}$ using the spectroradiometer. The results are significantly better than those shown in Figure 11, and the estimation error of ${\left\|\hat{x}-x_{0}\right\|}^{2}$ is 0.05718.

Similar relationships between the estimates by the proposed method and the minimum norm estimates were obtained for the other rice grain samples. To investigate the transferability of the ridge parameter, we obtained the parameters for a different sample of the same type for rice grains. These experimental results suggest that the parameter values obtained for the same type of rice grains are similar, but strictly speaking, it is difficult to correctly recover the spectral distributions for different samples using a common value of the ridge parameter. As a result, the ridge parameters should be estimated sample by sample.

Furthermore, we rendered the visual appearance of the fluorescent emission from the entire rice grains as an image. The human color perception is limited to the visible wavelength range. To determine the perceptual color of the fluorescence emission, we first applied the CIE color matching functions to the estimated fluorescence spectrum at each pixel point to obtain the tristimulus values XYZ in the visible wavelength region and then converted them into sRGB values.

**Figure 14:**
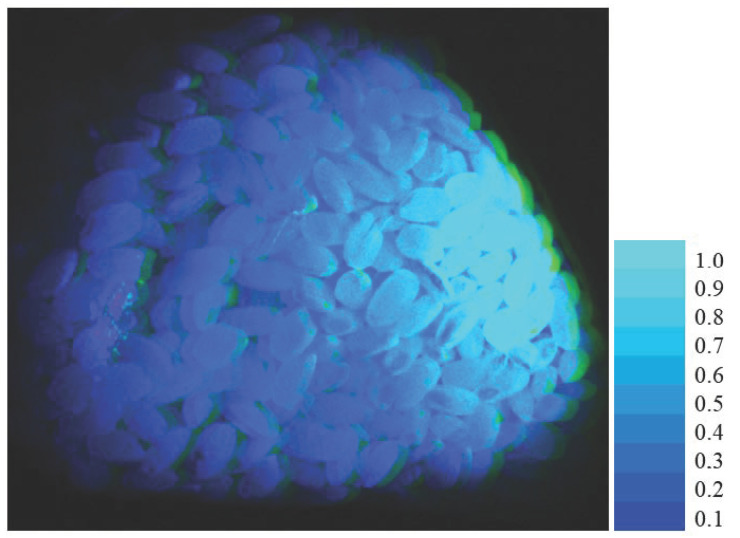
Visual appearance of fluorescence emission rendered with an sRGB image for the rice grain object. In the figure, the [0, 1] scale represents the relative intensity, where 1.0 is the maximum value. The gray areas are where no excitation light was illuminated and no fluorescence was emitted.

Figure 14 shows the visual appearance of the fluorescence emission rendered using the sRGB image of the rice grain object. In this figure, the scale [0, 1] represents the relative intensity, where 1.0 is the maximum value. The gray areas are those where no excitation light was illuminated, and no fluorescence was emitted.

### 4.2. Fluorescence Estimation for Plant Leaves

**Figure 15:**
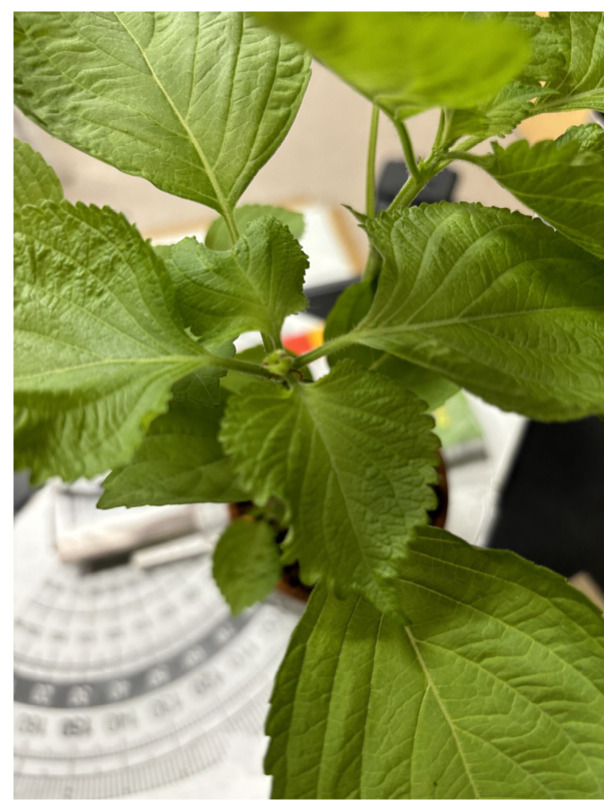
Photographic image of leaves “Ohba” of a living plant in a pot.

**Figure 16:**
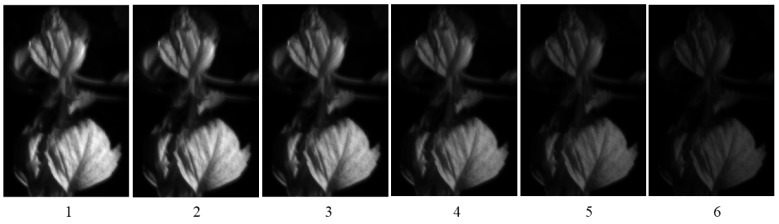
Images of each channel observed using the imaging system with six bands for the Ohba leave.

The images of the leaves “Ohba” of a living plant in a pot, as shown in Figure 15, were captured using the second imaging system. Ohba refers to the leafy part of “Green Shiso” (Perilla frutescens var. crispa), which is edible and sold on the market. Multiple images of Ohba leaves were obtained by sequentially changing the optical filters, which were then combined into a six-dimensional multiband image. Figure 16 shows an image of each channel observed using the imaging system with six bands.

**Figure 17:**
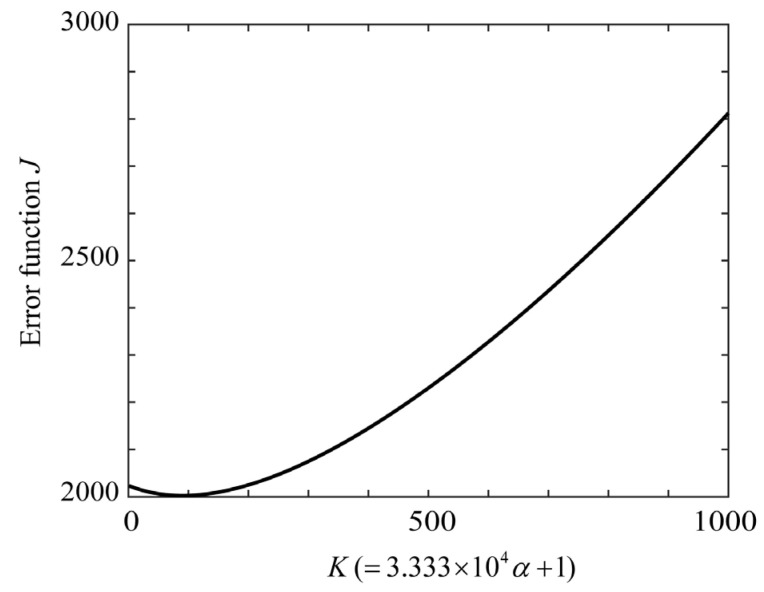
Error function *J* with *K* as a parameter to evaluate the ridge estimate for the plant leaves.

**Figure 18:**
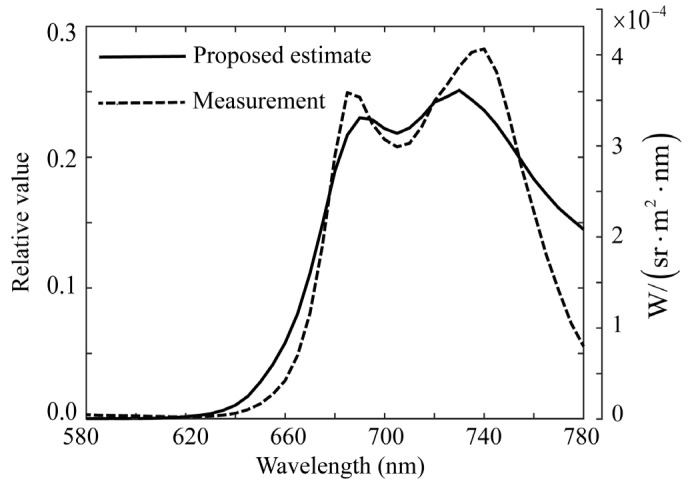
Ridge estimation result for the plant leaves, where the estimated spectral curve is compared with the direct measurement using the spectroradiometer. The scale in the right represents the physical quantities measured by the spectroradiometer.

The proposed ridge method was used to estimate the spectral distribution of the fluorescence emitted from the plant leaves. Because the fluorescence emission from plant leaves has a spectral distribution in the red-to-far-red wavelength regions, spectral estimation was performed in the wavelength region of 580–780 nm. Figure 17 shows the error curve of function *J*, where the parameter *K* is related to the ridge parameter, as in $\alpha \;=\;3.0\times 10^{-5}(K-1)$ (*K* = 1, 2., …, 1000). The *K* value and ridge parameter α that minimized *J* were *K* = 90 and α = 0.00267, respectively. Figure 18 shows the ridge estimation results, where the estimated $\hat{x}$ is compared with the direct measurement $x_{0}$. The estimation error of ${\left\|\hat{x}-x_{0}\right\|}^{2}$ is 0.03294. The scale on the right represents the physical quantities measured using a spectroradiometer.

**Figure 19:**
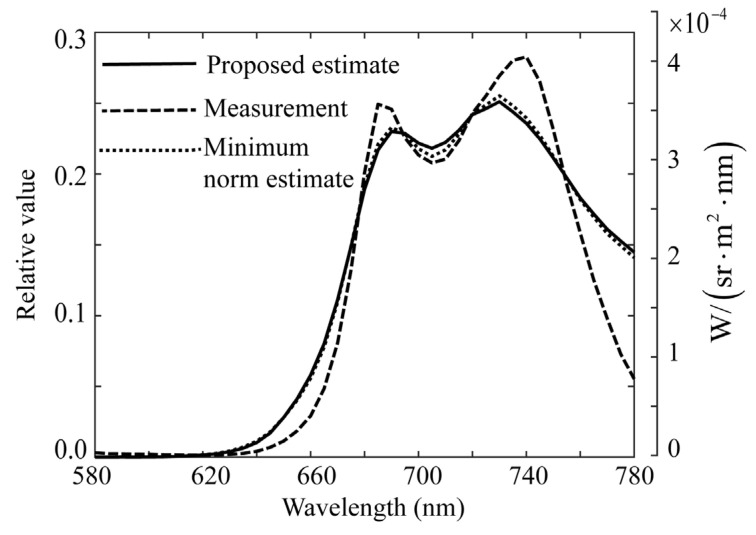
Comparison of three spectral curves between the ridge estimate, direct measurement, and minimum norm estimate for the plant leaves.

In addition, we examined the performance of the estimate using the minimum norm solution. Figure 19 compares the three spectral curves of the ridge estimate, direct measurement, and minimum norm estimate. The two spectral curves of the ridge and minimum norm estimates almost coincide; therefore, in this case, the minimum norm estimate exhibits good performance. Similar relationships between the estimates by the proposed method and the minimum norm estimates were obtained for the other plant leaf. As with the rice, to investigate the transferability of the ridge parameter, we obtained the parameters for a different sample of the same type for plant leaves. As a result, the parameter values obtained for the same type of plant leaf were similar, but strictly speaking, it was difficult to recover the spectral distributions for different samples using a common value of the ridge parameter value.

**Figure 20:**
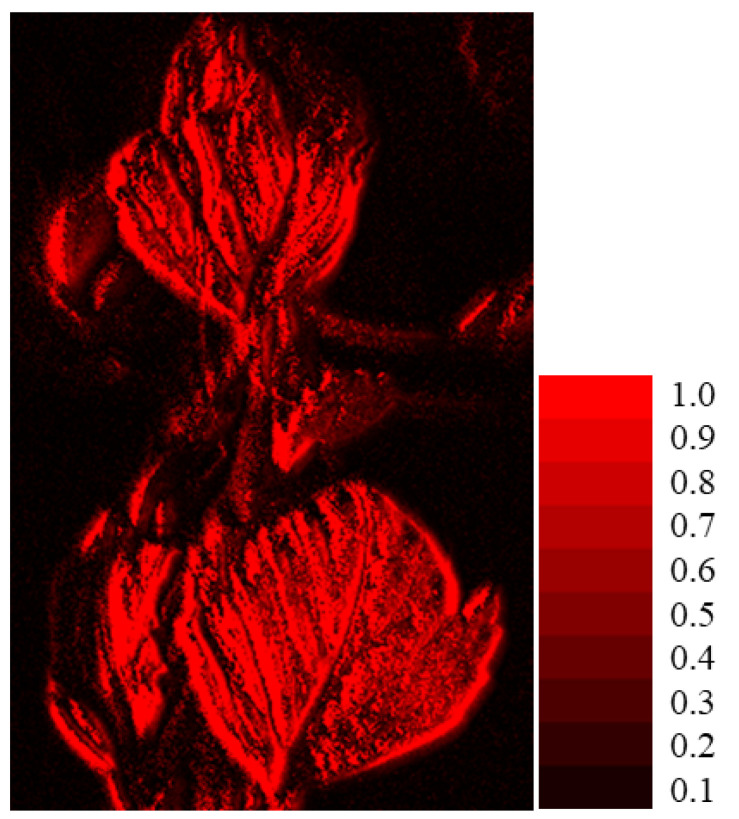
Visual appearance of fluorescence emission rendered with an sRGB image for the Ohba leave, where the [0, 1] scale represents the relative intensity with the maximum value 1.0.

Furthermore, we rendered the visual appearance of the fluorescence emission from the entire object of the Ohba leaf as an image. The perceptual color was calculated as the sRGB value at all pixel points in the visible wavelength region. Figure 20 shows the visual appearance of the fluorescence emission rendered by the sRGB image with the fluorescent color of the leaf, where the [0, 1] scale represents the relative intensity, with a maximum value of 1.0.

## 5. Conclusions

This paper has proposed a method for estimating the spectral images of fluorescence spectral distributions emitted from plant grains and leaves without using a spectrometer. Two types of multiband imaging systems with six channels were constructed by adding the minimum necessary optical filters to ordinary, small off-the-shelf cameras: one for rice grains by making a mobile phone camera multiband to detect the fluorescence emission in the visible wavelength region and the other for plant leaves using a monochrome camera with additional optical filters to detect chlorophyll fluorescence in the red-to-far-red wavelength region.

We adopted a ridge regression-based method to obtain a reliable estimate of the fluorescence emission spectral distribution at each pixel point using only the acquired image data. The fluorescence emission spectra can be estimated by optimally selecting the ridge parameters without using any statistics from the fluorescence spectra. An algorithm for optimal selection of this parameter was developed using cross-validation.

In the experiments using blended rice and Ohba leaves, UV light illuminated the plant targets and the emitted fluorescence images were captured using imaging systems. The fluorescence emission spectra of the rice grains and Ohba leaves were estimated using the proposed algorithm on the image data. These estimates were compared with direct measurements using a spectroradiometer and those estimated using the minimum norm solution method. In the former case, the proposed method was superior to the minimum norm method, whereas in the latter case, the estimation results of the proposed method and those of the minimum norm method were almost identical. Thus, the reliability of the proposed estimation method was confirmed. Furthermore, the visual appearance of fluorescence emission from the respective objects of the rice grains and Ohba leaves was rendered using sRGB images.

It should be noted that the estimation method for the fluorescence emission spectra proposed here is not limited to foods and plants, such as grains and leaves, but can be applied to the problem of estimating the emission spectra from any fluorescent object, including natural and artificial objects.

Our future work will include several studies that identify the origin of rice grains, evaluate their freshness, and investigate the growth and conditions of plant leaves.

## Data Availability

Data are available on request from the authors.
